# Solid-State Electrolytes for Lithium–Sulfur Batteries: Challenges, Progress, and Strategies

**DOI:** 10.3390/nano12203612

**Published:** 2022-10-14

**Authors:** Qiancheng Zhu, Chun Ye, Deyu Mao

**Affiliations:** School of Mechanical and Automotive Engineering, Guangxi University of Science and Technology, Liuzhou 545006, China

**Keywords:** lithium–sulfur battery, solid electrolyte, polymer electrolyte, inorganic solid electrolyte, composite electrolyte

## Abstract

Lithium–sulfur batteries (LSBs) represent a promising next-generation energy storage system, with advantages such as high specific capacity (1675 mAh g^−1^), abundant resources, low price, and ecological friendliness. During the application of liquid electrolytes, the flammability of organic electrolytes, and the dissolution/shuttle of polysulfide seriously damage the safety and the cycle life of lithium–sulfur batteries. Replacing a liquid electrolyte with a solid one is a good solution, while the higher mechanical strength of solid-state electrolytes (SSEs) has an inhibitory effect on the growth of lithium dendrites. However, the lower ionic conductivity, poor interfacial contact, and relatively narrow electrochemical window of solid-state electrolytes limit the commercialization of solid-state lithium–sulfur batteries (SSLSBs). This review describes the research progress in LSBs and the challenges faced by SSEs, which are classified as polymer electrolytes, inorganic solid electrolytes, and composite electrolytes. The advantages, as well as the disadvantages of various types of electrolytes, the common coping strategies to improve performance, and future development trends, are systematically described.

## 1. Introduction

With the continuous development of science and technology, new energy vehicles, portable electronic devices, and energy storage systems have resulted in higher requirements for the energy density of secondary batteries [1]. At present, the energy density of lithium–ion batteries is close to the limit of theoretical calculation. It is challenging for the energy density to break 400 Wh kg^−1^, even with advanced material design [2]. LSB is a prospective next-generation electrochemical energy storage system due to its high theoretical energy density of 2570 Wh kg^−1^, low price, and environmental friendliness [3].

Although lithium–sulfur batteries have many advantages, there are still some problems that hinder their commercialization: (1) the volume effect of the positive sulfur electrode in the process of charge and discharge within a volume expansion about 80% [4]; (2) the shuttle effect caused by the dissolution of the intermediate [5]; (3) the low conductivity of sulfur (10^−7^~10^−30^ S cm^−1^ at room temperature) [6]; and (4) the inhomogeneous lithium metal deposition caused by current density [7]. These problems accelerate battery aging and capacity decay. To suppress these problems, researchers have adopted a variety of strategies, such as developing various materials as sulfur carriers [8], introducing an intermediate layer as a physical barrier for chemisorption [9], improving the conversion efficiency of polysulfide [10], and modifying the biphasic interface [11]. Although these strategies have made some progress, they do not solve the root of the problem. Many researchers have turned their attention from flammable organic electrolytes to safer and more energy-dense solid electrolytes. Solid electrolytes can effectively avoid the shuttle effect induced by long-chain polysulfides. At the same time, the higher thermal stability of solid electrolytes avoids potential risks such as leakage or high-temperature flatulence, significantly improving the safety of batteries [12].

As the core part of a solid-state lithium–sulfur battery, the solid electrolyte dramatically affects battery performance. A good SSE must have the following characteristics: (1) A high ion mobility number is required, and when the ion mobility number is low, the cell will have severe local polarization, resulting in uneven Li^+^ deposition and lithium dendrite generation [13]. (2) Higher Li-ion conductivity: Low ionic conductivity means that the battery has higher internal resistance, which also reduces the charge and discharge rate of the battery [14]. (3) A stable electrochemical window: The ideal SSE electrochemical window should be stable between 0 and 5 V [15]. (4) Good flexibility ensuring mechanical strength: The mechanical strength can inhibit the penetration of lithium dendrites, and the flexibility makes the interface between electrode and electrolyte more fit and stable [16]. (5) Good electrical insulation [17]: Until now, a single SSE has not been able to cover all of these features. Different types of SSEs are applied in different situations according to actual requirements and to face different challenges. In recent years, several reviews on the SSEs of Li–sulfur batteries have been reported. However, these reviews only involve the respective challenges and solutions of different SSEs, but few reviews systematically describe the challenges faced by SSEs and the respective characteristics of different SSEs. Therefore, before describing the research progress made regarding SSEs, this paper describes the challenges faced by SSE and the differences between different SSE in detail, which is helpful to deepen readers’ understanding of different SSEs. In addition, this paper also summarizes some feasible or novel strategies and predicts directions of future development based on the existing research results.

## 2. Principles and Challenges of Lithium–Sulfur Batteries

### 2.1. The Composition and Working Principle of Lithium–Sulfur Battery

**Figure 1:**
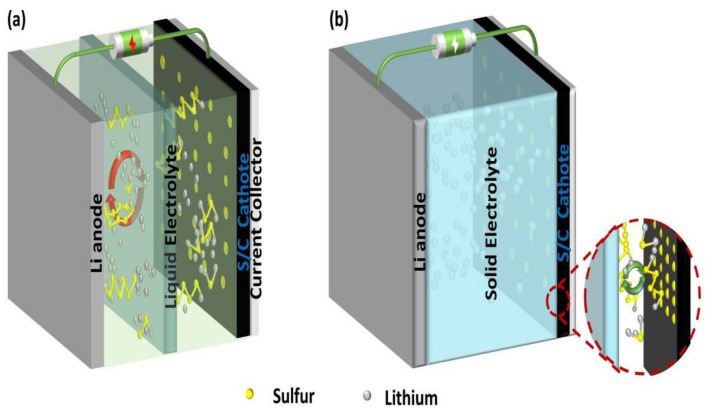
The schematic structure of (**a**) liquid-electrolyte and (**b**) solid-electrolyte lithium–sulfur battery.

A typical Li–sulfur battery system consists of a sulfur cathode, a lithium metal anode, and an electrolyte. Unlike the de-embedded lithium energy storage mechanism of traditional lithium-ion batteries, LSBs consist of a reversible redox reaction between lithium metal and S_8_ for the mutual conversion of chemical energy and electric energy. The entire reaction process can be expressed by the equation S_8_ + 16Li → 8Li_2_S [18]. During the discharge process, the solid ring S_8_ gradually becomes the long liquid chain polysulfide Li_2_S_n_ (4 < n ≤ 8) and the short-chain polysulfide Li_2_S_n_ (2 < n ≤ 4), which are finally converted to insoluble Li_2_S_2_ and Li_2_S. As shown in Figure 1a, in a liquid electrolyte one, both long-chain polysulfides and short-chain polysulfides are soluble in the electrolyte, so the shuttle effect inevitably occurs. LiPS shuttles back and forth in the electrolyte and reacts directly with lithium metal in the negative electrode, causing the irreversible loss of active substances and capacity in the battery. The shuttle effect is a common problem in liquid Li–sulfur batteries and is the main reason for capacity decay. As shown in Figure 1b, a solid electrolyte without liquid components can prevent LiPS from moving to the anode, thus realizing the enhancement of LSB cycle stability and Coulomb efficiency.

### 2.2. The Challenges of Lithium–Sulfur Solid Electrolytes

The biggest problem facing conventional liquid-electrolyte lithium–sulfur batteries is the shuttle effect of lithium polysulfide. The shuttle effect is the phenomenon in which LiPS dissolves in the electrolyte and shuttles back and forth between the two electrodes when the battery is being used. LiPS reacts with lithium metal in the shuttle process, which dramatically reduces the capacity and cycle stability of Li–sulfur batteries, causing severe and damaging corrosion to the anode. There are two commonly used solutions. One is to introduce functional materials into the cathode or electrolyte and to fix polysulfide on the surfaces of the functional materials through the physical barrier or chemisorption [19]. The other is to insert an interlayer to prevent polysulfide diffusion [20]. Unfortunately, these measures only suppress but do not entirely solve the shuttle effect. A practical solution is to use solid electrolytes. The solid-phase reaction of a solid electrolyte can directly transform S into Li_2_S, which effectively avoids LiPS dissolution and the shuttle effect, and its charge and discharge curve only shows a voltage plateau. Although the solid electrolyte effectively avoids the shuttle effect, the solid-phase reaction also faces the challenge of slow kinetics.

At present, SSEs can be roughly divided into three types according to the different materials: polymer electrolyte (PE), inorganic solid electrolyte (ISE), and composite solid electrolyte (CSE). The problems and challenges faced by several types of solid-state lithium–sulfur batteries include the low ionic conductivity of the solid-state dielectric, interface incompatibility, poor chemical/electrochemical stability, and lithium dendrite growth.

#### 2.2.1. Low Ionic Conductivity

Ion conductivity (σ) is a critical index that determines the internal resistance and multiplier performance of a battery. SSEs with excellent performance should have high ionic conductivity to realize the rapid transport of lithium ions. At the same time, it has electronic insulation to avoid the growth of lithium dendrites and the occurrence of self-discharge. The room-temperature ion conductivity of most SSEs is still low compared to liquid electrolytes, resulting in the poor electrochemical performance of the cells and limiting the development of SSLSB. The polymer electrolyte is easy to manufacture and is flexible enough to form good interfacial contact with the anode and cathode. The most common mechanism of lithium-ion transport in polymer electrolytes is segmental motion, which is characterized by the coordination and dissociation of migrating ions and polymer groups under the action of the electric field. Most ion transport occurs in the amorphous region, and the crystallization state at room temperature leads to low ionic conductivity. Generally, ionic conductivity is improved by reducing the crystallinity of polymers, manifested as polymer blending and copolymerization. Compared to solid-polymer electrolytes, inorganic solid electrolytes have higher ionic conductivity, but inorganic solid electrolytes are brittle, which leads to the SSLSB having high interface contact [21]. To some extent, inorganic solid electrolytes and polymer electrolytes can complement and combine to form inorganic–organic composite electrolytes. Compared to the single electrolyte mentioned above, the inorganic–organic composite electrolyte has higher ionic conductivity and a lower glass transition temperature.

#### 2.2.2. Interface Incompatibility

Improving interface compatibility has always been a research hotspot in the SSE field. Compared to flexible gel polymer electrolytes, the interface incompatibility between the solid electrolyte and electrode are more prominent. Among them, the oxide SSE is more rigid, and the interface contact area between the electrode and SSE is limited. The interface problem is severe and even causes a serious polarization phenomenon and slow kinetics. Improvement strategies are generally divided into two aspects: increasing the contact area using porous and multilayer structures designed by deposition technology or manufacturing by selecting an intermediate buffer layer comprising appropriate materials [22]. Unlike oxide SSEs, sulfide SSEs are relatively soft and have good interface contact with the cathode and anode. The interface problem of sulfide SSE is mainly due to the space charge layer effect caused by the limited electrochemical window and the insulation of sulfur. To avoid the impact of the space charge layer, interface coating is a decent strategy.

#### 2.2.3. Chemical/Electrochemical Stability

Chemical stability refers to the ability of the battery to resist chemical reactions in the idle state to maintain stable physical and chemical properties. Electrochemical stability refers to the ability of the battery to maintain physical and chemical properties under the action of an external electric field [23]. LSBs often use lithium as the anode, which is relatively reactive and easy to react with SSE, resulting in chemical/electrochemical instability. Among the common SSEs, solid polymers are relatively stable with lithium metal, and sulfide-based SSEs are less electrochemically stable. Sulfide-based SSs have become the SSEs with the best potential due to its their ionic conductivity and low interfacial impedance. However, sulfide-based SSEs are extremely sensitive to water and unstable to lithium metal, which significantly limits the application of sulfide-based SSEs in batteries with a high-energy-density power batteries [24]. Adding a coating material at the interface between the SSE and the electrode represents an excellent method for surface optimization. The coating material can provide surface passivation protection and prevent the diffusion of non-lithium elements at the interface. When the thickness of the coating material is lower than that of the decomposition product layer, the interface resistance can also be effectively reduced.

#### 2.2.4. Lithium Dendrite

Lithium dendrites are mainly caused by inhomogeneous deposition at the Li/electrolyte interface [25]. Uncontrolled Li dendrite growth can cause serious safety problems. Researchers hope to prevent the growth and penetration of lithium dendrites via inorganic solid electrolytes with high mechanical strength. With the development of characterization techniques, researchers have found that although the high mechanical strength of inorganic SSE could theoretically resist dendrite growth, Li dendrites still appear in the bulk phase of inorganic SSEs, eventually penetrating the SSE and causing short circuiting in the battery [26]. On the one hand, the crystal structure of inorganic SSE electrolytes promotes the growth of Li dendrites. Once there is a gap, Li dendrites will penetrate the inorganic SSE, grow along the grain boundary, and gradually penetrate the positive pole [27]. On the other hand, the high electronic conductivity of SSE promotes Li^+^ binding to electrons in the electrolyte and the gradual formation of Li dendrites. There are currently many strategies for inhibiting Li dendrites, but all of them have some defects. It is necessary to deepen our understanding of the mechanism of lithium dendrite growth to provide a possible way to inhibit Li dendrite effectively.

## 3. Polymer Electrolytes

At present, common matrix materials for polymer solid electrolytes include polyethylene oxide (PEO) [28], polyvinylidene fluoride (PVDF) [29], polyacrylonitrile (PAN) [30], polyvinylidene fluoride hexafluoropropylene (PVDF-HFP) [31], polymethyl methacrylate (PMMA) [32], and its derivatives [33]. Compared to inorganic solid electrolytes, polymer-based SSEs have better flexibility, can adapt to changes in the positive electrode volume, and provide good interface contact. Good processing properties make them easy to manufacture have more conducive to commercial development. However, polymer-based SSEs usually have low ionic conductivity, poor mechanical properties, and are prone to the shuttle effect.

### 3.1. Gel Polymer Electrolyte/Quasi Solid Electrolytes

A gel polymer electrolyte (GPE) is a polymer matrix body that ensures mechanical strength and a liquid electrolyte that provides electrochemical properties that allow the formation of a gel-state electrolyte. GPEs are characterized by high ionic conductivity because their lithium-ion transport mechanisms are similar to those of liquid ones (>10^−3^ S cm^−1^) [34]. However, because the most commonly used electrolyte in GPEs is ether solvent, the solubility of LiPS in an ether electrolyte will lead to the shuttle effect, and the flammability of ether electrolyte will also increase the safety risks of batteries.

#### 3.1.1. PVDF Base

**Figure 2:**
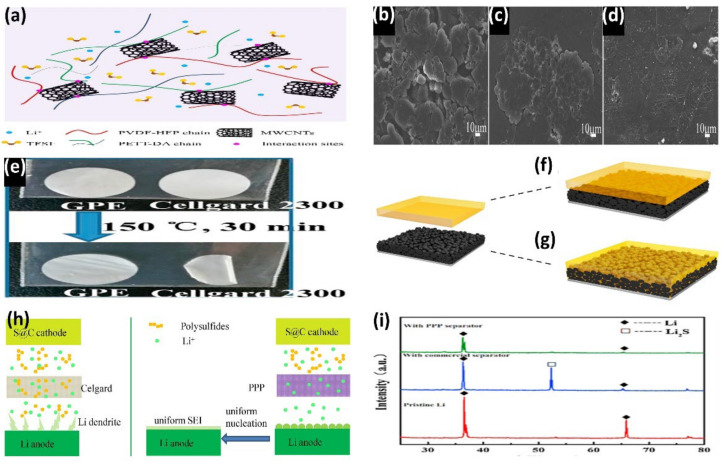
(**a**) Schematic of UPHC 3D polymer network formed by intermolecular hydrogen bonding effect (Reprinted with permission from [36]; Copyright 2020, Chemical Engineering Journal). SEM image of Li anode in Li symmetric cell after 20 cycles of plating/stripping with different electrolytes at a current density of 0.5 mA cm^−1^. (**b**) Celgard; (**c**) PVDF; (**d**) MOF-PVDF (Reprinted with permission from [39]; Copyright 2019, ACS Applied Materials & Interfaces). (**e**) Comparison of 3D GPE network and Celgard 2300 diaphragm before and after heat treatment at 150 °C for 30 min (Reprinted with permission from [40]; Copyright 2019, Chemical Engineering Journal). Formation diagram of the electrode–electrolyte interface. (**f**) The GPE acts directly as a film between the electrodes. (**g**) GPE-impregnated electrodes (Reprinted with permission from [41]; Copyright 2017, ACS Applied Materials & Interfaces). (**h**) Schematic diagram of Li and polysulfide diffusion differences between Celgard- and PPP-membrane-assembled LSB during charging and discharging (Reprinted with permission from [42]; Copyright 2020, Electrochimica Acta). (**i**) XRD patterns of pristine Li; the Li after 50 cycles at 200 mA g^−1^ with the commercial separator and with the PPP separator (Reprinted with permission from [43]; Copyright 2016, Electrochimica Acta).

PVDF is a common polymer framework in GPEs. This kind of electrolyte has the advantages of good wettability, easy film preparation, and a high dielectric constant, but it also faces the disadvantages of interface instability, poor cycling performance, among other problems. To overcome the problem of interface instability, Han et al. prepared a PVDF membrane with a simple phase transformation method and modified the PVDF membrane by self-polymerization with lithiophilic polydopamine (PDA). This lipophilic GPE can improve the stability of the lithium anode and allow it to form a dense and stable SEI film during long-term cycling. It can also inhibit the shuttle effect through the strong interaction with polysulfide, which dramatically enhances the cycle performance of the battery. PDA-PVDF provided an excellent Coulomb efficiency of more than 98% during the cycling process at a 0.1 C 200-turn cycle [35]. Wang et al. used pentaerythritol tetrakis–divinyl adipate (PETT-DA) as a 3D network matrix, long-chain polyvinylidene fluoride–hexafluoro propylene (PVDF-HFP) as co-doped flexible matrix, and MWCNTs as an interactive nanofiller to prepare a new type of 3D GPE film (denoted as UPHC) with superior comprehensive properties. PVDF-HFP has high ionic conductivity and excellent toughness. PETT-DA can inhibit the shuttle effect and the growth of Li dendrites. MWCNTs, as a filling material, can further increase the amorphous region and improve ionic conductivity. Different compositions play their respective functions and achieve sound synergistic effects by forming intermolecular hydrogen bonds, as shown in Figure 2a. After 300 cycles at 0.5 C, UPHC demonstrated excellent cycle stability, with a capacity retention rate of 86.4% (down from 704.5 mAh g^−1^ to 608.8 mAh g^−1^) and a Coulomb efficiency greater than 99.5% [36]. Metal–organic skeletons (MOFs) are widely used in GPE due to their high specific surface area, prosperous and orderly porous structure, and promotion of lithium-ion conduction [37,38]. Han et al. reported a novel PVDF-based GPE modified by MG-MOF-74, which can fix large polysulfide anions, trap anions in pores, and promote the uniform flux of Li and the uniform deposition of Li to stabilize the lithium anode. SEM was performed on the lithium surface of the new GPE after 20 cycles. As shown in Figure 2b–d, MG-MOF-74-based PVDF electrolytes have a smooth lithium surface, which indicates that the MG-MOF-74 material has a uniform lithium plating/stripping process [39]. It is an essential requirement that electrolytes have high thermal stability for the commercialization of LSB. Xia et al. prepared GPE with a 3D network using PVDF-HFP as a matrix with the assistance of simple ultraviolet light. As shown in Figure 2e, when the electrolyte was heat-treated at 150℃ for 30 min, GPE only showed minimal changes and high thermal stability compared to Celgard 2300, which suffered severe coiling and shrinkage [40].

#### 3.1.2. PEO Base

PEO-based GPEs have the advantages of low cost, simple manufacturing, and good electrochemical stability. Because ion transport mainly occurs in the amorphous region, the high crystallinity at room temperature results in low ionic conductivity. In addition, the flammability of PEO itself also dramatically increases the safety risk of the battery. PEO can be modified by copolymerization, blending, crosslinking, and doping. Increasing the safety of battery use and inhibiting its crystallization improves the ion conductivity.

Sheng et al. reported a poly (ethylene oxide)–polyacrylonitrile (PEO-PAN) copolymer membrane electrolyte, and PAN acts as both a filler and cross-linker. This structure can not only improve ionic conductivity, improve mechanical strength, and inhibit the growth of lithium dendrites. It can also prevent LiPS shuttle and improve the cycle performance of batteries via the C = N-O functional groups formed during the cross-linking process between PEO and PAN [44]. Han et al. developed an electrode impregnation and pre-crosslinking GPE (cPT-A-Bs) with a large ionic liquid (IL)-absorption capacity. The precursor of cPT-A-Bs is composed of hydrophilic, hydrophobic, and hydrophilic triblock (PEO-PPO-PEO), which is interlinked by the sol-gel process. The triblock precursors are mixed with different amounts of IL to produce gel GPE films and solid GPE films. The electrodes were impregnated with gel precursors to form an excellent electrode–electrolyte interface (Figure 2f,g). The electrolyte containing 200 wt% IL showed good ionic conductivity (5.0 × 10^−3^ S cm^−1^) and good mechanical stability (maximum strain = 194%) [41]. Due to its low lattice energy, PEO can be used in highly dissolved lithium metal salts and provide Li channels. Besides being used as a matrix, PEO is often used as an additive to improve the performance of polymer electrolytes [45]. Wang et al. proposed a new 3D ultraviolet polymeric electrolyte. A PVDF-HFP nanofiber membrane (PPP membrane) modified by PEO was inside the electrolyte. The hydrogen bond between PEO and PVDF-HFP can improve electrochemical performance by adjusting the diameter of the nanofiber and the porosity of the membrane. The ultraviolet-polymerized pentaerythritol tetrakis–divinyl adipate (PETT-DA) layer serves as the outer coating, and the carbonyl group in it can make up for the deficiency of PVDF-HFP films in inhibiting LiPS shuttling. Figure 2h shows the differences in LiPS and Li diffusion during cycling for LSB assembled with Celgard and PPP membranes. The assembled LSB had a capacity retention rate of 87.1% (from 543 mAh g^−1^ to 473 mAh g^−1^) after 300 cycles at 2 C [42].

#### 3.1.3. PMMA Base

The strong polarity of the carbonyl group can form a strong connection with carbonate, which makes PMMA have a strong electrolytic affinity. It helps more electrolytes to be absorbed, enhancing the ionic conductivity of the electrolyte. In addition, PMMA has good electrochemical stability and low interfacial impedance [46]. However, PMMA has a lower lithium-ion transfer number (<0.5) and a fragile matrix, making commercialization difficult. Copolymerization and blending are common methods to improve the performance of PMMA.

Shi et al. prepared a PEO/PMMA/PVDF-HFP gel polymer matrix using a co-blending method. The matrix exhibited an ionic conductivity of 8.1 × 10^−4^ S cm^−1^ at 25 °C and good interfacial stability with the lithium metal anode [47]. Kang et al. prepared a new GPE mixed with PEO, PS, and PMMA using solvent-pouring technology. Through polymer blending, the synergistic advantage of different polymer materials can make up for the deficiencies of a single polymer matrix. After testing, the room temperature ion conductivity of the new GPE is 1.2 × 10^−4^ S cm^−1^, resulting in the Li metal electrode having high electrochemical stability and good interface stability [48]. To inhibit polysulfide shuttling, Yang et al. constructed a PVDF/PMMA/PVDF (PPP) sandwich GPE. The outer porous PVDF can absorb the ether electrolyte to improve the lithium-ion transfer rate. The inner solid PMMA layer is compatible with the ether electrolyte and can capture polysulfides well. The lithium metal was analyzed after 50 cycles using XRD patterns, as shown in Figure 2i, and no new crystalline phase formation was found, which proved that polysulfide shuttling was further weakened.

In summary, currently commonly used polymer substrates have their advantages and disadvantages, which cannot fully meet commercialization needs. Among them, poor interface stability, low ion conductivity, electrochemical instability, and the shuttle effect are the main factors inhibiting its commercialization. The instability of the PVDF interface can be modified by using a lithiophilic material. The common ionic conductivity of PEO and the low mechanical properties of PMMA can be improved by copolymerization or by blending various polymers. The molecular arrangements of PEO and PMMA can be destroyed by polymer blending or copolymerization, thus reducing their crystallinity. However, as the number of polymer substrates used for blending or copolymerization increases, it becomes more difficult to choose a plasticizer that is compatible with many polymers simultaneously. In addition, conventional organic plasticizers such as carbonates and ethers may increase the hazards during battery use. The ether oligomers or flame-retardant polymers such as ethyl phosphate–polyethylene glycol-based copolymers (EPCP) can be used or introduced into GPEs.

### 3.2. Solid Polymer Electrolytes

Solid polymer electrolytes (SPEs) are composed of polar polymers and lithium electrolyte salts. Compared to GPEs, they can completely remove the liquid components and have the advantages of being non-flammable, easy to process, high mechanical strength, and good chemical/electrochemical stability. However, the ionic conductivity of SPE under room temperature is low. The standard method to improve the ionic conductivity of SPE is to increase the temperature and to reduce the crystallinity of the polymer. With the increased temperature, the SPE will gradually soften, and the dissolution of LiPS will be accelerated, causing a severe shuttle effect.

**Figure 3:**
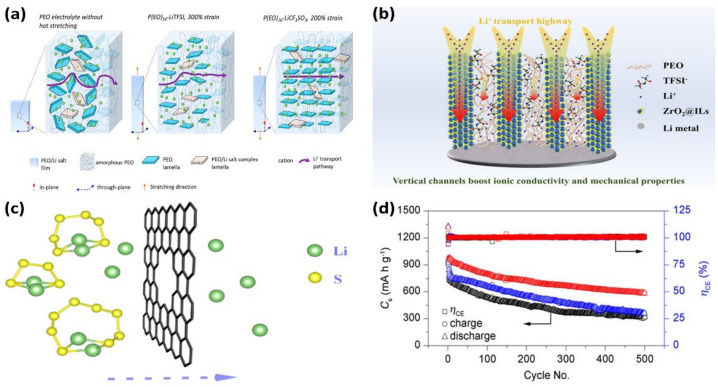
(**a**) Schematic diagram of different electrolyte lamellar orientations and lithium-ion transport pathways (Reprinted with permission from [49]; Copyright 2022, ACS Macro Letters); (**b**) PEO polymer with ZrO_2_@ILs vertical channel design to improve mechanical strength and ionic conductivity (Reprinted with permission from [50]; Copyright 2021, ACS Applied Materials & Interfaces). (**c**) Schematic diagram of polysulfide and lithium-ion transport by graphene-coated PP diaphragm (Reprinted with permission from [53]; Copyright 2018, ACS Applied Materials & Interfaces). (**d**) Electrochemical performance diagram of UIO-66-modified PP diaphragm and contrast sample (Reprinted with permission from [54]; Copyright 2019, ACS Omega).

Providing a fast method for ion transfer is a common way to improve ionic conductivity. Liu et al. reported a hot drawing technique by which crystalline sheets perpendicular to the drawing direction were selected, and the crystallinity of the electrolyte was reduced. As shown in Figure 3a, this enhanced ionic conductivity in the through-plane direction of the SPE films by reducing the ionic conduction pathway [49]. Liang et al. combined the zirconia skeleton (ZrO_2_@ILs) with the vertical channel of fast Li ion movement generated by hydrolysis in situ with PEO to enhance the conductivity and mechanical properties of electrolyte ions, as shown in Figure 3b. Compared to conventional PEO, the ionic conductivity of PEO-based electrolytes with fast vertical channels increased from 10^−6^ S cm^−1^ to 10^−4^ S cm^−1^, and the lithium-ion migration number rose from 0.19 to 0.48 [50]. Wan et al. demonstrated a polymer–polymer solid electrolyte with a polyimide (PI) film with high mechanical strength as the main body and PEO/LiTFSI as the ion transport filler. The high modulus of PI film can increase the possibility of mechanical force to inhibit the penetration of lithium dendrites, and the vertical channel helps to improve ionic conductivity. Compared to traditional PEO-based SPE, the ionic conductivity of the PEO-based SPE with the PI vertical channel was improved at all measured temperatures [51]. Wang et al. proposed a novel method to enhance the ionic conductivity of electrolytes using dynamic covalent bonding. The incorporation of disulfide bonds and oligomeric ethylene oxide-based additives (S-S additives) into PEO-based polymers demonstrates high ionic conductivity of 1.24 × 10^−4^ S cm^−1^ at room temperature [52].

The physical barrier or chemisorption of an electrolyte to LiPS is a common strategy to inhibit the shuttle effect [55]. Wang et al. prepared a novel dual-function polymer electrolyte: PDDA-TFSI-PVDF-HFP (PTP), via an anion exchange reaction. The adsorption between PDDA-TFSi and LiPS can effectively alleviate the shuttle effect of LI-S cells. Moreover, the interaction between TFSI− and quaternary ammonium ions in PTP was used to obtain a higher mobility number of lithium ions [56]. Zhuo et al. designed a novel bilayer polyoxyethylene polymer electrolyte to limit LiPS. The electrolyte layer on the negative side is composed of pure PEO, and on the positive side, on the basis of PEO, a PVP inhibition shuttle effect with strong affinity for LiPS is added [57]. Ou et al. first applied a single layer of graphene on the polypropylene film (PP film) as an intermediate layer and then used the interfacial polymerization process to seal the larger holes in the graphene. As shown in Figure 3c, the graphene nanopore structure can block the migration of LiPS while maintaining the transport of lithium ions, thus weakening the shuttle effect. Compared to the original PP film, the initial specific capacity of the PP film coated with graphene was increased from 983.2 mAh g^−1^ to 1128.4 mAh g^−1^ at 0.05 C. The coulomb efficiency was also increased from 96.0% to 99.9%, which significantly increased the battery capacity and cycle stability [53]. Fan et al. reported a UiO-66-modified PP diaphragm, which significantly improved the cycling stability of LSB. The narrow and uniformly distributed particle size of UiO-66 crystals can physically block LiPS, and the enhanced chemical interaction with polysulfide can be used for chemisorption. As shown in Figure 3d, the capacity of the modified PP diaphragm remains at 586 mAh g^−1^ after 500 cycles at 0.5 C, and the average capacity retention rate is calculated as 99.85%, showing good cycle stability [54].

Low ionic conductivity at room temperature and shuttle effects are the biggest obstacles to SPE application. Increasing the temperature in use is a common strategy to improve ionic conductivity, but this is often accompanied by severe shuttle effects. Building fast ion transport channels or enhancing ion conductivity through copolymerization are suitable methods to improved ion conductivity. Using the complex decomposition of covalent bonds to assist lithium-ion transport is also a novel and promising method, but there is still relatively little research in this area. For the shuttle effect, the physical barrier or chemisorption ability of LiPS can be enhanced by SPE modification or the application of an intermediate layer. It should be noted that when chemisorption is performed, the amount of dissolved polysulfide in the electrolyte gradually increases as S loading or the cycle number increase, which can lead to slow reaction kinetics. By introducing a catalyst on the electrolyte surface, the energy conversion barrier of polysulfides can be reduced, and polysulfide reuse can be enhanced.

## 4. Inorganic Solid Electrolytes

Compared to polymer-based electrolytes, inorganic solid electrolytes (ISEs) have higher mechanical strength, which can inhibit short circuits caused by lithium dendrite penetration and can effectively avoid the shuttle effect that causes polysulfide insolubility. It is worth mentioning that the wide electrochemical window of ISE can be matched with high-voltage positives to obtain a higher battery energy density. Currently, the most widely studied ISE is oxide inorganic solid electrolytes and sulfide inorganic solid electrolytes.

### 4.1. Oxide Base

Oxide-based ISEs can mainly be divided into garnet-type Li_7_La_3_Zr_2_O_12_ (LLZO) electrolytes, perovskite-type Li_3x_LA_2/3-x_TiO_3_ (LLTO) electrolytes, NASICON-type Li_1+X_ Al_x_Ti_2−X_(PO_4_)_3_(LATP) electrolytes, Li_1+X_Al_x_Ge_2−X_(PO_4_)_3_ (LAGP) electrolytes, etc. Compared to polymer electrolytes, oxide-based ISEs exhibit higher ionic conductivity and have good stability with the high-voltage positive electrode and lithium-metal negative electrode. Unfortunately, the brittle nature of oxide-based ISEs and the significant interfacial resistance between the electrode and electrolyte have largely prevented their commercialization.

**Figure 4:**
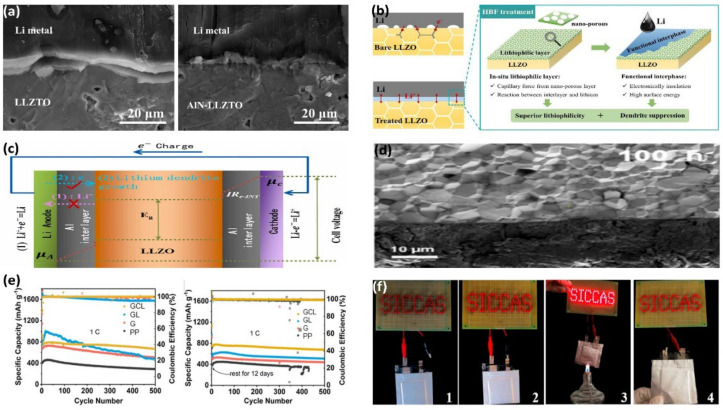
(**a**) SEM images of Li/LLZTO interface and ALN-LLZTO interface (Reprinted with permission from [58]; Copyright 2022, Nanomaterials (Basel)). (**b**) Schematic diagram of the interface between pure LLZO and LLZTO-Hb anode and description of the main functions of the lithophilic layer and the interface formed (Reprinted with permission from [59]; Copyright 2022, Energy Storage Materials). (**c**) Lithium dendrite inhibition mechanism of Li-conducting and electron-non-conducting Li-Al-O interlayers (Reprinted with permission from [61]; Copyright 2022, Journal of Alloys and Compounds). (**d**) Out-of-situ SEM images of Li/Zn-Cu-LlZto-Zn-Cu/Li after 100 h cycling at 0.8 mA·cm^−2^ (Reprinted with permission from [62]; Copyright 2021, ACS Applied Materials & Interfaces). (**e**) The initial cycling performance of PP, G, GL, and GCL LSB at 1 C and the cycling performance after 12 days (Reprinted with permission from [63]; Copyright 2022, ACS Applied Materials & Interfaces). (**f**) LSB battery in (1) open, (2) connected, (3) burning, and (4) broken LED light (Reprinted with permission from [64], Copyright 2019 Energy Storage Materials).

LLZO is the most widely studied oxide-based ISE. It has high ionic conductivity (10^−3^–10^−4^ S cm^−1^ at room temperature) and a wide electrochemical stability window. Poor interface stability and lithium dendrite growth are the problems that need to be solved. Introducing an intermediate layer between Li and LLZO is a standard method to improve interface contact. Jiang et al. introduced an aluminum nitride (AlN) middle layer between lithium anode and LLZO to improve the interface contact problem. The AlN interlayer is lithiophilic and can wet the electrolyte interface and fill the pores on its surface well. This helps to increase the close contact between the electrode and the electrolyte, facilitating the diffusion of lithium ions. As shown in Figure 4a, there is apparent undesirable contact and a large void between the lithium metal without introducing the interlayer and the LLZTO electrolyte. The introduction of the AlN interlayer fills the cavity on the surface of the solid electrolyte, resulting in close contact between the lithium electrode and LLZTO [58]. As shown in Figure 4b, Cai et al. constructed a lithium-salt layer with a nanostructure and that was lithiophilic on the surface of LLZO through HBF treatment. The reaction between the lithium-salt layer and Li can improve the wettability between LLZO and molten lithium and reduce the interfacial impedance. The interfacial layer generated by the response can block not only the electron transport but also guide the uniform deposition of lithium [59]. Huang et al. used Li–Nafion to coat the surface of LLZO with a dense ionic conductive layer to inhibit the shuttle effect. The Coulomb interaction between the conducting layer and the sulfonate group can effectively prevent LiPS shuttle and allows lithium-ion conduction, which improves the cycling performance of Li–sulfur batteries [60].

To explore the mechanism of lithium dendrite growth, Zhang et al. constructed an Al metal interlayer with electronic conductivity and an Al_2_O_3_ interlayer with electronic insulation on the Li/LLZO interface by magnetron sputtering and annealing. The results show that lithium dendrites are more easily formed on the Al metal intermediate film with electron conductivity. The electrons can directly combine with Li through the intermediate layer to form lithium dendrites. Li-Al-O compounds with Li conductivity and electronic insulation are generated by Al_2_O_3_ and Li in the charge–discharge cycle, as shown in Figure 4c. Their electronic insulation can hinder the conduction of electrons, inhibit the growth of lithium dendrites, and promote the lithium deposition reaction [61]. To inhibit the growth of lithium dendrites, He et al. designed a Cu-doped Li_3_Zn bifunctional composite layer on LLZO by magnetron co-splashing and in situ alloying reactions. The introduction of the mixed layer can effectively reduce the interfacial impedance, and Cu nanoparticles can be used as a conductive layer to help the uniform deposition of lithium. After cycling at 0.8 mA cm^−2^, Li dendrites appeared on the interface after only 35 h of pure Zn modification. As shown in Figure 4d, the Cu-doped group still maintains good interface contact after 100 h. It is worth noting that since Cu is a poor conductor of Li, when the amount of Cu doped in the Zn layer is large (Zn:Cu > 1:5), the resistance will increase significantly [62].

Although LLTO electrolytes have high electrochemical stability and a high elastic modulus, their use is limited by high interfacial resistance. It is necessary to reduce interfacial impedance and improve ion conduction. Wang et al. coated an LLTO/SP composite layer on the commercial polyethylene membrane (Celgard) diaphragm. The modified diaphragm showed lower interfacial impedance and a higher ion diffusion coefficient and significantly inhibited the shuttle effect [65]. Zhu et al. proposed a composite bilayer framework, which coupled the one-dimensional LLTO/PEO composite electrolyte with the 3D CNF/S cathode. The addition of LLTO enlarges the amorphous region of the PEO and provides a continuous ion transport channel. The double-layer structure between the electrolyte and cathode reduces the interfacial impedance and enhances the interfacial stability [66].

LATP and LAGP are the two most popular research systems for NASICON ISE. They are obtained by partially replacing Ti^4+^ (or Ge^4+^) by Al^3+^, which has a smaller ionic radius and has the advantages of high ionic conductivity and electrochemical stability. Taking advantage of the strong LiPS adsorption capacity and superior Li ion conductivity of LATP, Xiao et al. combined LATP with a Ti_2_(SO_4_)_3_/carbon protective layer (CLATP) with graphene to construct a multifunctional intercalation layer (GCL) with high ionic conductivity as well as high electronic conductivity. As shown in Figure 4e, after 12 days of standing, the battery using GCL has no capacity loss, and it still offers a discharge capacity of 676 mAh g^−1^ after 500 cycles at a 1 C current. The capacity decay of each cycle is only 0.022%. It has incredibly high self-discharge performance and cycle stability [63]. To solve the interface problem of ISE, Li et al. introduced a CPE buffer layer and conductive graphene layer into the double interface modification between ISE and the anode and cathode, respectively. LGAP eliminates the shuttle effect, the graphite layer can provide redox sites to improve sulfur utilization, and the CPE layer can reduce the LAGP reduction effect of the lithium anode. As shown in Figure 4f, the soft-package Li-S battery modified for the LATP interface can light the LED lamp under the conditions of raw, broken, and burning, showing the great potential of double-interface modification in ISE [64].

The inherent mechanical stiffness of ISE inhibits the shuttle effect, but it can also form large interface impedance and easily fracture during the manufacturing process. The use of an LLTO-based ISE is limited by the high interfacial impedance and the instability of Ti^4+^ elements to lithium metal. LLZO is far more stable to lithium than other solid electrolytes, but it is easy to produce inert lithium carbonate, which leads to interface problems and lithium dendrite growth. Therefore, an effective solution for the interlayer is introduced. Due to the instability of Ti^4+^ and Ge^4+^ to lithium, LATP and LAGP as electrolytes easily produce side reactions and increase the resistance of the interface. It is an excellent method to add an interface protective layer or to use organic compounds to compound with them. Oxide ISEs are one of the few ISEs that can be used with lithium metal as well as high-voltage cathodes, which helps to increase the energy density of the battery. The introduction of an intermediate layer will cause it to lose this advantage, and reducing the thickness of the electrolyte is a possible trend for future development.

### 4.2. Sulfide Base

Compared to oxide-based ISEs, sulfide-based ISEs have higher ionic conductivity and better interface contact due to the weak electronegativity, sizeable ionic radius, and lower mechanical strength of sulfur. In addition, sulfide-based ISEs also have the advantages of easy cold-forming and good compatibility with the sulfur cathode, indicating good application prospects in LSBs. However, the relatively narrow electrochemical window leads to easy oxidation via the positive electrode and reduction caused by the negative electrode. The poor interfacial electrochemical stability increases the interfacial impedance and promotes the growth of lithium dendrites. Li_10_GeP_2_S_12_ (LGPS) and Li_6_PS_5_Cl (LPSCl) are the two most widely studied sulfide ISE systems.

**Figure 5:**
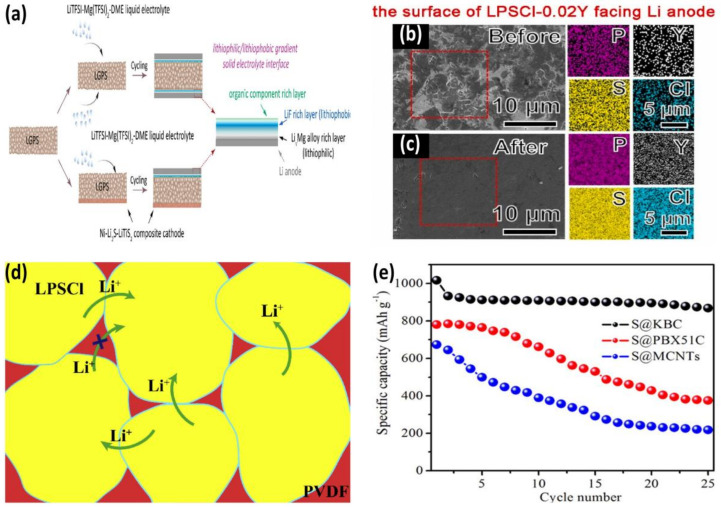
(**a**) In situ formation of LixMg/LiF/polymer and the solid electrolyte interface between Li and LGPS (Reprinted with permission from [67]; Copyright 2021, ACS Energy Letters). (**b**) The SEM image and EDS mapping results of the interface between solid electrolyte and Li anode after 100 cycles are (**b**) LPSCl−0.02Y before 100 cycles and (**c**) LPSCl−0.02Y, respectively (Reprinted with permission from [68]; Copyright 2022, Journal of Power Sources). (**d**) Schematic diagram of the microstructure of LPSCl/PVDF composite electrolyte (Reprinted with permission from [69]; Copyright 2020, Journal of Materiomics). (**e**) Cycling performance of PYR_13_TFSI−modified LGPS in different sulfur/carbon composite LSB electrodes (Reprinted with permission from [70]; Copyright 2019, Journal of Materiomics).

To cope with the reduction of LGPS and the growth of lithium dendrites, Wan et al. introduced a composite phase layer between the electrolyte and the lithium anode (Figure 5a). The LiF interlayer is widely used between LGPS and a negative Li electrode because of its low electronic conductivity, high lithium-specific surface energy, and good mechanical strength. In addition, the introduction of lithiophilic LixM (M = metal) and flexible polymers can greatly reduce the possible increase in interfacial impedance caused by LiF [67]. Xia et al. doped Y^3+^ in LPSCl electrolytes. Due to the expansion of the PS_4_^3−^ lattice and the increased solubility of Li in the LPSCl crystal structure, the ionic transport capacity of the electrolyte was enhanced, and LPSCl-0.02Y exhibited higher ionic conductivity than the original LPSCl in the range of 25–100 ℃. In addition, as shown in Figure 5b,c, the surface of the LPSCl-0.02Y electrolyte layer is still flat and compact after 100 cycles, offering excellent interfacial compatibility [68]. Wang et al. introduced PVDF polymer into the LPSCl electrolyte (Figure 5d). They used PVDF to fill the gaps between the LPSCl particles, thus improving the cycling stability between the LPSCl electrolyte and lithium anode [69]. Umeshbabu et al. explored a simple and precise method using lithium bis(trifluoromethanesulfonyl)imide (LiTFSI)/N-methyl-N-propylpyrrolidinium bis(trifluoromethanesulfonyl)imide (PYR_13_TFSI) as a surface modifier to provide an ion conduction network and to enhance the stability of the interface between LGPS and a lithium anode. As shown in Figure 5e, an initial capacity of 1068 mAh g^−1^ and a good capacity retention rate are displayed when used in conjunction with the positive sulfur electrode in S@KBC [70]. Zhao et al. enhanced the stability of the negative lithium electrode via the fluoridation of theLi_6_PS_5_Cl electrolyte. The fluorinated sulfide electrolyte shows good durability and diversity in LMB, which provides us with a new strategy [71].

The good mechanical properties and excellent room-temperature ion conductivity are the reasons for the wide attention paid to sulfide-based ISEs. The instability of the interface with a lithium anode, the growth of lithium dendrites, and the sensitivity to water vapor are the obstacles facing the development of sulfide-based ISEs. The stability between the electrolyte and electrode can be improved by introducing a protective layer or electrolyte fluoridation. In addition, the sulfide-group ISEs can be easily hydrolyzed to produce toxic H_2_S, and the inert preparation environment dramatically increases production costs. The sensitivity of sulfide-based ISEs to water vapor can be improved by metal oxide doping or by a composite with a hydrophobic polymer, and the prospect of its commercial application can be improved. Although O instead of S can enhance sulfide ISE stability, the stronger binding of lithium ions to oxygen is detrimental to the dissociation of lithium salts, and a balance between excellent stability and high ionic conductivity is required in practical applications.

## 5. Composite Electrolytes

Generally speaking, a CPE is a composite electrolyte with low interfacial impedance and high lithium-ion conductivity, which is possible via the introduction of inorganic fillers into organic polymers to exert the synergistic effect of the two. The addition of inorganic fillers can reduce the crystallinity of the polymer and increase the amorphous region of ion transport. In addition, the higher mechanical strength of inorganic fillers helps to increase the mechanical properties of polymer materials and improve the ability of electrolyte film formation. According to how they conduct ions, inorganic fillers can be divided into inert and active fillers.

### 5.1. Inert Fillers

**Figure 6:**
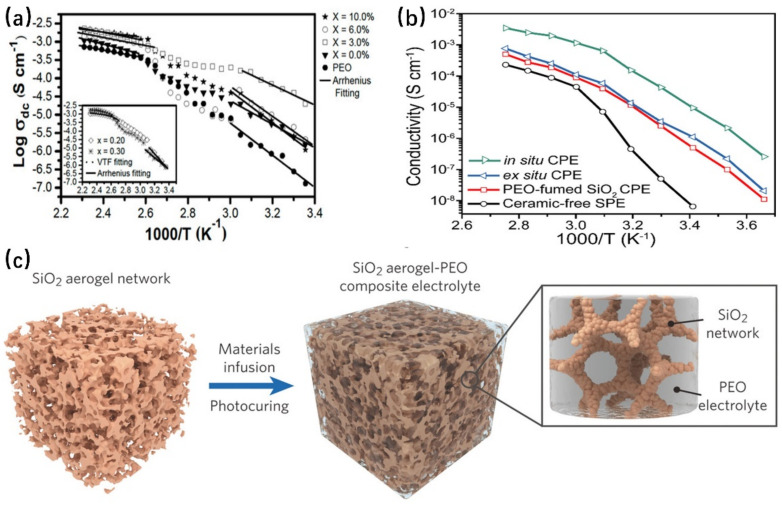
(**a**) The conductivity of the (PEO)_10_CF_3_COONa polymer electrolyte. The (PEO)_10_CF_3_COONa+x wt.% Al_2_O_3_ composite electrolyte is inversely proportional to temperature. Solid lines represent Arrhenius fit, and dashed lines represent Vogel−Tammann−Fulcher (VTF) fit (Reprinted with permission from [72]; Copyright 2019, Materials (Basel)). (**b**) Arrhenius diagram of ionic conductivity between the PEO composite electrolyte containing SiO_2_ and the contrast sample (Reprinted with permission from [74]; Copyright 2017, Powder Technology). (**c**) Schematic diagram of the synthesis process of aerogel skeleton silica and PEO (Reprinted with permission from [75]; Copyright 2018, Advanced Materials).

Inert fillers cannot conduct ions, and the mechanism is generally to increase ionic conductivity by inhibiting the crystallization of the polymer. Inert filler is generally an oxide, such as SiO_2_, TiO, Al_2_O_3_, and so on. Delgado et al. tested the polymer membrane of polyethylene oxide (PEO) and sodium trifluoroacetate (PEO: CF_3_COONa) combined with different concentrations of alumina (Al_2_O_3_) particles. The crystallinity of the polymer varied with the concentration of Al_2_O_3_ particles, and the sample with an Al_2_O_3_ concentration of 3.0% had the lowest crystallinity. As shown in Figure 6a, it has the highest ionic conductivity at this time [72]. Lee et al. coated the surface of polyethylene PE film with α-Al_2_O_3_ powder of very high purity that was prepared by jet milling as well as hydrothermal methods. Compared to the wet ball mills, the purity of the spray mill was 99.997%. When overlaid on the surface of PE, the diaphragm exhibits high absorbance (243–245 wt%) and porosity (60–63%). At high temperatures, the enthalpy value and shrinkage rate of the composite electrolyte with the Al_2_O_3_ coating were significantly reduced [73]. Lin et al. introduced an in situ synthesis method for ceramic particles, which made use of the solid interaction between SiO_2_ nanospheres generated by in situ hydrolysis and the PEO chain to reduce the crystallinity of PEO. As shown in Figure 6b, the in situ hydrolysis technique further promoted the improvement of ionic conductivity compared to the pure PEO polymer electrolyte and the composite electrolyte with mechanically mixed PEO and SiO_2_ [74]. Lin et al. introduced SiO_2_ with an aerogel skeleton structure into the PEO polymer, as shown in Figure 6c. The SiO_2_ aerogel framework increases the mechanical properties of the composite electrolyte. The elastic modulus is two orders of magnitude higher than that of the traditional polymer electrolyte, which is about 0.43 GP at room temperature. In addition, the large internal surface area of the SiO_2_ structure promotes the interaction between lithium and anions, and the ionic conductivity reaches 6 × 10^−4^ S cm^−1^ at room temperature [75].

### 5.2. Active Filler

Active filler has the ability to transport ions and can be used as a solid electrolyte alone, making it more advantageous than inert materials. Currently, typical active materials include LATP, LAGP, LLTO, LGPS, etc.

**Figure 7:**
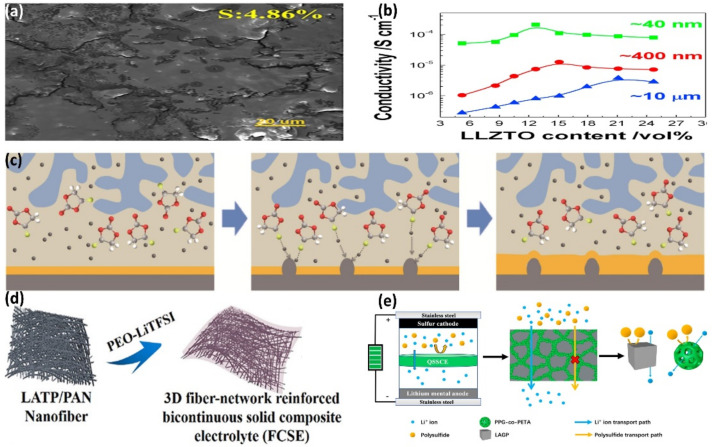
(**a**) SEM image of PEO−Pan−LLZO CPE 1C lithium anode after 500 cycles (Reprinted with permission from [78]; Copyright 2022, Chemical Engineering Journal). (**b**) The conductivity of LLZTO particles with different sizes varies with the volume fraction of LLZTO (Reprinted with permission from [79]; Copyright 2016, Nano Energy). (**c**) Interface self-healing process of LLTO−(PEO−FEC) (Reprinted with permission from [80]; Copyright 2021, Chemical Engineering Journal). (**d**) Schematic diagram of LATP/PAN 3D skeleton and CPE structure of PEO (Reprinted with permission from [81]; Copyright 2018, ACS Applied Materials & Interfaces). (**e**) schematic diagram of PPG−Co−PETA/LAGP CPE for suppressing polysulfide shuttle effect in LI-S cells (Reprinted with permission from [82]; Copyright 2021, Materials (Basel)).

Liu et al. prepared a PTFE@LLZO@PEO composite electrolyte with PEO as a polymer matrix, PTFE film as a support structure, and Li_7_La_3_Zr_2_O_12_ as the inorganic filler. The addition of PTFE not only increases the mechanical properties of the electrolytes, but also improves its thermal stability and ionic conductivity. After the introduction of LLZO particles and PTFE skeleton, PEO exhibited a significant decrease in the intensity of two characteristic diffraction peaks at 19° and 23° (2θ), which showed the interaction between PTFE, LLZO, and PEO, increasing the amorphous region of PEO [76]. Xu et al. introduced a Li_7_La_3_Zr_2_O_12_ ceramic nanonetwork into the PEO/TPU/LiTFSI matrix at different concentrations via the electrospinning method to study the effects of different concentrations of LLZO on the performance of composite electrolytes. The results showed that the composite electrolyte with 10 wt% LLZO possesses the highest ionic conductivity (1.33 × 10^−3^ S cm^−1^ at 60 ℃), which was three times higher than that of the pure PEO/TPU/LiTFSI electrolyte. In addition, benefiting from the vast and stable electrochemical window of LLZO, the electrochemical window width of the composite electrolyte reaches 5.6 V [77]. Xie et al. used liquid-phase synthesis to prepare LLZO–PAN–PEO dispersions and acetylene black–PVDF dispersions, which were coated on both sides of the nonwoven fabric to cope with the common shuttle effect and lithium dendrite growth in GPE. The interfacial compatibility of PEO–PAN–LLZO with lithium anodes was good and facilitated uniform lithium electroplating/stripping. The porous carbon layer itself could effectively absorb LiPS and inhibit the shuttle effect. The conductive acetylene black coating on the membrane effectively hindered LiPS migration and enhanced redox kinetics as an electronic conductor and physical barrier. As show in Figure 7a, the composite electrolyte lithium anode is structurally quite smooth after 500 cycles at 1 C. This indicates that a uniform and stable SEI film is formed on the surface of the lithium anode, which effectively inhibits the growth of lithium dendrites [78]. Zhang et al., prepared LLZTO with different particle sizes via high-energy ball milling to explore the relationship between particle size and the ionic conductivity of LLZTO/PEO composite electrolyte. As shown in Figure 7b, compared to the micron-sized composite electrolyte, the ionic conductivity of the composite electrolyte with an LLZTO particle size of 40 nm is increased by two orders of magnitude. This is mainly attributed to the large surface area of small particles, which increases the coherent conductive path [79].

Because a small number of fast ionic conductor nanomaterials can provide continuous ion transport routes, Zhu et al. introduced LLTO nanowires into the PEO matrix to prepare composite electrolytes. With a 5 wt% LLTO nanowire content, the composite electrolyte shows 5.53 × 10^−5^ S cm^−1^ ionic conductivity at room temperature. In addition, the introduction of LLTO nanowires improved the electrochemical stability of the composite electrolyte [83]. Kou et al. designed an asymmetric LLTO framework with porous and dense layers and impregnated it into PEO to prepare composite electrolytes. The LLTO framework provides a rapid ion transport pathway for the composite electrolyte. The thick layer promotes the uniform deposition of Li and improves the mechanical strength of the electrolyte, which helps to inhibit the growth of lithium dendrites [84]. Li et al. prevented the development of lithium dendrites by introducing additive fluoroethylene carbonate (FEC) into the LLTO/PEO composite electrolyte. As shown in Figure 7c, when the interface is damaged by lithium dendrites, the FEC decomposes under the coordination action of FEC-Li at the damaged interface to form a new LiF-rich layer to encapsulate the lithium dendrites and to repair the interface damage. Different from the LLTO/PEO composite electrolyte, which was penetrated by lithium dendrites after 520 h, the composite electrolyte with FEC remained stable after 800 h of lithium plating/stripping, which provided a new idea for inhibiting lithium dendrites [80].

Xia et al. simply prepared composite electrolytes by homogeneously filling LATP as an inorganic filler into a PVDF-HFP polymer. The composite electrolyte has a high ion transfer number of 0.51 and exhibits an initial discharge capacity of 918 mAh g^−1^ at 0.05 C when applied to lithium–sulfur batteries [85]. As shown in Figure 7d, Li et al. used LATP/PAN as a 3D skeleton and combined it with a PEO-based polymer to prepare a 3D fiber network composite electrolyte with good flexibility as well as high stability. LATP-loaded PAN fibers can improve the mechanical strength of PEO polymers and inhibit the growth of lithium dendrites. At the same time, the PAN coating helps to enhance the electrochemical stability of the interface by avoiding direct contact between LATP and lithium anode. The composite electrolyte maintained excellent electrochemical stability after 15 days of contact with lithium metal [81]. Since vertically ordered inorganic particles can promote ionic conductivity well, Wang et al. prepared vertically arranged LAGP using the ice template method and combined it with the PEO. Under room temperature, the ionic conductivity of the vertically arranged LAGP composite electrolyte reaches 1.67 × 10^−4^ S cm^−1^, six times that of disordered LAGP [86]. Deng et al. made a composite electrolyte (PPG-Co-PETA/LAGP) from a blended composite material comprising poly (propylene glycol) -co-pentaerythritol triacrylate and Li_1_._5_Al_0_._5_GE_1_._5_(PO_4_) using the situ violet curing method. The composite electrolyte with different LAGP contents was characterized. When the LAGP content was 66.85 wt%, the ionic conductivity and amount of lithium-ion migration in the composite electrolyte reached maximum levels of 5.95 × 10^−3^ S cm^−1^ and 0.83, respectively. The addition of LATP reduced the mesoporous size of the composite electrolyte and effectively blocked the polysulfide shuttle in LSB, as shown in Figure 7e. In addition, the initial discharge capacity of LSB at 0.25 C reached 1508.1 mAh g^−1^, and the capacity retention rate was 73.6% after 200 cycles, showing good electrochemical cycling performance [82].

The composite electrolyte combined the advantages of both organic polymers and inorganic fillers to improve the performance of LSB. Compared to inert fillers that cannot transport ions, active fillers that can transport ions are more advantageous. Reducing the particle size of inorganic fillers, introducing continuous ion transport channels, and an orderly arrangement of inorganic fillers are suitable methods to improve ionic conductivity. In addition, the inorganic fillers with skeletons can improve the mechanical strength of electrolytes while improving the ionic conductivity, inhibiting the growth of lithium dendrites, or physically blocking the polysulfide shuttle. It is important to note that inorganic fillers have strong surface effects, and when the inorganic filler content exceeds a threshold value, the agglomeration of fillers or the insulation of inactive fillers can have a great impact on the ionic conductivity of the composite electrolyte. Interfacial modification of the filler can reduce the surface energy of the filler and enhance the dispersion of the filler in the polymer matrix.

## 6. Conclusions and Prospects

As the requirements for battery energy storage and safety performance continue to increase, solid-state lithium–sulfur batteries have become a research hotspot in the field of energy storage due to their high safety and high energy density. Among them, solid electrolytes have been discussed because they inhibit the shuttle effect and lithium dendrites in addition to their high safety. This review starts with a basic description of the fundamentals of LSBs and the challenges faced by SSEs. Finally, it introduces the latest research progress and common strategies for SSEs. The performance of electrolytes were summarized in Table 1, and the development of the three type electrolyte were compared in Table 2.

Polymer-based SSEs have high flexibility, can buffer the volume expansion of sulfur cathode well, and make good contact the electrode. However, their low ionic conductivity at room temperature and the LiPS shuttle effect seriously hinders their practical application. Structural modifications to polymers or inorganic fillers are usually adopted to improve the above problems. The glass transition temperature of SSEs can be reduced by blending, copolymerization, cross-linking, etc., to suppress the overall crystallinity of the material and thus increase the ionic conductivity. However, as the polymer matrices increase, higher requirements are put forward for plasticizer selection. The addition of inorganic fillers can improve the mechanical strength of the electrolyte. In addition, the ionic transport ability of the active inorganic filler itself and Lewis’ acid–base reaction with the polymer can effectively contribute to ionic conductivity.

Compared to GPE, the inorganic solid electrolyte has higher ionic conductivity and better thermal stability, which can effectively avoid the shuttle effect caused by LiPS. The wide electrochemical window of an inorganic solid electrolytes can be used as high-voltage cathode materials to further improve battery energy density. However, due to the rigidity of inorganic materials, the contact between anode and cathode makes the interface resistance more significant, greatly reducing the cycling performance of inorganic materials. Interface modification or the introduction of a buffer layer are standard treatment methods. It should also be noted that the oxide ISEs are prone to form Li_2_CO_3_ on the surface when used, and sulfide ISEs are prone to reacting with water to form toxic H_2_S. These issues can be solved by heat pulse, acid treatment, and metal oxide doping on the surface of ISEs.

Currently, neither pure inorganic solid electrolytes nor polymer-based electrolytes can meet the performance requirements of Li–sulfur batteries. Polymer electrolytes have low room temperature ionic conductivity, and inorganic solid electrolytes with high ionic conductivity also face problems such as high interfacial impedance. Composite SSEs with both polymers and inorganic fillers are an effective method to improve battery performance. The solid-polymer electrolytes have good flexibility and higher interface compatibility with positive and negative electrodes. The higher mechanical strength of inorganic solid electrolytes can inhibit the growth and puncture of lithium dendrites.

Due to the effort invested by researchers, lithium–sulfur batteries have made great breakthroughs in SSE, but there are still some problems that still restrict the commercialization of SSLSBs, and the following aspects still need to be focused on: (1) Improving ionic conductivity: The ionic conductivity of composite electrolytes still represents a gap that prevents application, and low ionic conductivity reduces the advantages of the high specific energy LSBs. It is necessary to study the mechanism of ion transport in depth, to try new materials continuously, and to find the suitable concentration of each material as well as the most appropriate treatment method. (2) Optimizing the electrolyte/electrode interface structure: A battery system with excellent performance requires a good compatibility between the anode, electrolyte, and cathode and the construction of a stable ion transport network to enable fast and uniform Li transport. At present, there are many studies on flexible sulfur cathodes, but there are fewer studies on flexible Li cathodes, and there is a need for further research on flexible Li cathodes. (3) Considering the battery-use environment, there is a lack of research on electrolytes under low and high temperature-use conditions, which is essential research. In general, the key mechanisms of ion transport and the lithium dendrite growth mechanism in solid-state batteries need to be explored in depth in the future, and new stable interface structures need to be constructed. Extreme use environments also need to be considered. In addition, the existing manufacturing process also needs to be improved to enhance the performance of Li-S solid-state batteries from both theoretical and practical perspectives.

## Figures and Tables

**Table 1:** Performance of electrolytes with different composites.

Composites	Type	Ionic Conductivity (S cm^−1^)	Initial Capacity (mAh g^−1^) (Current Density)	End of Capacity (mAh g^−^^1^) (Cycle Number)	Reference
PDA–PVDF	GPE	/	1215.4 (0.1 C)	868.8 (200)	[35]
PET-DA/PVDF-HFP/MWCNTs	GPE	1.1 × 10^−3^	704.5 (0.5 C)	608.8 (300)	[36]
Mg-MOF-74/PVDF	GPE	6.72 × 10^–4^	996.7 (1 C)	778.4 (250)	[39]
PVDF-HFP/PETT/Ester monomer	GPE	6.61 × 10^–4^	601 (0.5 C)	326.94 (300)	[40]
PEO/PVDF-HFP/PET-DA	GPE	9.64 × 10^−4^	543 (2 C)	473 (300)	[42]
PVDF/PMMA/PVDF	GPE	1.95 × 10^−3^	1173.6 (1000 mA g^−1^)	523.1 (300)	[43]
PEO/PAN/LiTFSI	GPE	1.63 × 10^−5^	1200 (0.1 C)	803.1 (75)	[44]
LiPF_6_/FEC/PE	GPE	/	930 (0.5 C)	800 (800)	[87]
PEO/PFA	SPE	/	1128.4 (0.1 C)	798.6 (20)	[53]
UiO-66/PVDF	SPE	/	1032 (0.5 C)	586 (500)	[54]
P(VDF-HFP) (PDDA-TFSI-P(VDF-HFP)	SPE	1.76 × 10^−3^	1241 (0.2 C)	813 (200)	[56]
PEO-10%HUT4/LiTFSI	SPE	8.2 × 10^−6^	640 (1 C)	498 (500)	[13]
Graphene/Ti_2_(SO_4_)_3_/Li_1_._3_Al_0_._3_Ti_1_._7_(PO_4_)_3_	Oxide based ISE	3.09 × 10^–4^	779 (1 C)	671 (500)	[63]
LLAZO/LLAZO/CNF	Oxide based ISE	2.51 × 10^–4^	1055 (0.2 C)	939 (50)	[88]
LiTFSI/PYR_13_TFSI/Li_10_GeP_2_S_12_	Sulfide based ISE	2.04 ×10^–3^	1068 (83.5 mA cm^–2)^	868 (25)	[70]
PTFE/LLZO/PEO	CPE	5.03 × 10^−5^	655 (0.1 C)	568 (100)	[76]
PEO/PAN/LLZO	CPE	2.01 × 10^−3^	942 (1 C)	555 (500)	[78]
PPG-co-PETA/LAGP	CPE	5.95 × 10^−5^	1508 (0.25 C)	1109.5 (500)	[82]
PVDF-HFP/LATP	CPE	7.41 × 10^−4^	918 (0.05 C)	458.9 (40)	[85]
PVDF/LiClO_4_/LATP	CPE	8.07 × 10^−5^	620.52 (0.5 C)	202.3 (500)	[89]

**Table 2:** Advantages, challenges and common solutions of electrolytes.

Electrolyte Type	Advantages	Challenges	Strategies
Polymer electrolytes	Good flexibility Excellent interfacial compatibility Easy to process	Low ionic conductivity Lithium dendrite Poor thermal stability	Blending, copolymerization, cross-linking Construction of fast ion channels
Inorganic solid electrolytes	High ionic conductivity High voltage resistant Mechanical properties	Poor interface compatibility Brittle and cracked Poor air stability	Interface modification Introducing the middle layer Metal oxide doping
Organic-inorganic composite electrolyte	High ionic conductivity Suitable mechanical properties	Inorganic filler aggregation Complex operation process	Control packing concentration, particle size Modification of fillers

## Data Availability

Not applicable.
